# Modulation of Immunogenicity and Conformation of HA1 Subunit of Influenza A Virus H1/N1 Hemagglutinin in Tubular Immunostimulating Complexes

**DOI:** 10.3390/ijms18091895

**Published:** 2017-09-03

**Authors:** Nina Sanina, Ludmila Davydova, Natalia Chopenko, Eduard Kostetsky, Valery Shnyrov

**Affiliations:** 1 Department of Biochemistry, Microbiolgy and Biotechnology, Far Eastern Federal University, 690091 Vladivostok, Russia; l.vorobushek@mail.ru (L.D.); natali_1389@mail.ru (N.C.); kostetskiy.yeya@dvfu.ru (E.K.); 2 Departamento de Biochimica y Biologia Molecular, Universidad de Salamanca, 37008 Salamanca, Spain; shnyrov@usal.es

**Keywords:** TI-complex, adjuvant, antigen delivery system, monogalactosyldiacylglycerol, lipid-induced protein conformation, DSC, intrinsic fluorescence, vaccines

## Abstract

The HA1 subunit of the influenza virus hemagglutinin (HA) is a valuable antigen for the development of vaccines against flu due to the availability of most antigenic sites which are conformational. Therefore, a novel adjuvanted antigen delivery system, tubular immunostimulating complexes (TI-complexes) comprising monogalactosyldiacylglycerol (MGDG) from different marine macrophytes as a lipid matrix for an antigen, was applied to enhance the immunogenicity of recombinant HA1 of influenza A H1N1 and to study the relation between its immunogenicity and conformation. The content of anti-HA1 antibodies and cytokines was estimated by ELISA after the immunization of mice with HA1 alone, and HA1 was incorporated in TI-complexes based on different MGDGs isolated from green algae *Ulva lactuca*, brown algae *Sargassum pallidum*, and seagrass *Zostera marina*. Conformational changes of HA1 were estimated by differential scanning calorimetry and intrinsic fluorescence. It was shown that the adjuvant activity of TI-complexes depends on the microviscosity of MGDGs, which differently influence the conformation of HA1. The highest production of anti-HA1 antibodies (compared with the control) was induced by HA1 incorporated in a TI-complex based on MGDG from *S. pallidum*, which provided the relaxation of the spatial structure and, likely, the proper presentation of the antigen to immunocompetent cells.

## 1. Introduction

Viruses of *Influenzavirus A* genus, which belongs to the *Orthomyxoviridae* family, are the cause of all flu pandemics. The causative agent of the first and the most aggressive flu pandemic of the 20th century, the Spanish flu, which killed 20–40 million people worldwide in 1918–1919, was the Influenza virus A subtype H1N1. This subtype circulated in different variations among the global population until 1957. In 1977, H1N1, this time with very similar antigenic properties, appeared again, and has been recurring in the human population ever since. In 2009, a new influenza virus, which also was a subtype of H1N1 but differed antigenically from previously circulating viruses, spread rapidly around the world [1]. Molecular genetic studies have shown that the A (H1N1) pdm2009 Influenza Strain is a triple reassortant, carrying avian, human, and swine influenza virus A gene segments [2]. 

The prevention and global control of pandemic H1N1 are mainly implemented by the wide distribution of effective vaccines. The greatest expectations are related to the development of effective subunit vaccines (third-generation vaccines) containing only virus flu surface proteins and, therefore, differing from the whole-virion vaccines and split-vaccines by exhibiting low reactogenicity [3].

Hemagglutinin (HA) is the dominating membrane protein of the influenza virus A virions. HA molecules protrude above the surface of the virus particle. Therefore, it is no accident that subunit flu vaccines comprise HA, which is responsible for the interaction of the virus with the cell surface of a host organism and, consequently, for the neutralization of viruses [4].

Each monomer of homotrimeric HA is composed of two disulphide-linked subunits, HA1 (328 amino acids) and HA2 (221 amino acids), which arise from protein precursor (HA0) after cleavage by a host cell protease. HA1 is responsible for the binding to terminal sialic acid residues of receptors on the surface of the upper respiratory tract epithelial cells, which is the first stage of viral replication. All antigenic determinants of HA inducing the formation of neutralizing antibodies belong to the globular head of HA1 [5,6]. Moreover, antibodies to a highly conserved helical region in the membrane-proximal stem of HA1/HA2 are able to neutralize the virus infectivity by blocking membrane fusion. Therefore, the relevant epitope is considered as a target for the development of a universal influenza A vaccine [7,8].

However, proteins, either full-length or truncated, included in subunit vaccines are generally weak antigens. Hence, an adjuvant is needed to stimulate immune response to the protein antigen. Additionally, the conformation of such an antigen may be different from that in the virus capsid, which can lead to a change in the antigenic properties of the protein.

Tubular immunostimulating complexes (TI-complexes), which are self-organized from a mixture of three constituents (triterpene glycoside cucumarioside A_2_-2 from marine invertebrate *Cucumaria japonica*, cholesterol, and glycolipid monogalactosyldiacylglycerol (MGDG) from marine macrophytes) are a novel adjuvanted antigen delivery system [9]. Their glycolipid component forms a lipid matrix for the protein antigen and, therefore, can influence the conformation and antigenic properties of the protein incorporated in the TI-complex. 

Our previous studies have shown that TI-complexes can not only significantly enhance the immune response against porin of enterobacteria *Yersinia pseudotuberculosis*, but also allow for the optimization of vaccine construction by changing the physicochemical properties of the lipid surrounding the protein and, consequently, affecting its conformation and immunogenicity [10]. TI-complexes incorporating HA0 of Influenza A H1N1 hemagglutin (A/California/04/2009(H1N1) also stimulated the production of anti-HA0 antibodies [11]. However, the level of anti-HA0 antibodies did not depend on the physicochemical properties of MGDGs despite changes in the spatial structure of HA0. We assumed that conformational changes of HA0 were associated with the interaction of MGDG with the HA2 subunit, whose hydrophobic N-terminus forms a fusion protein of hemagglutinin [12]. Meanwhile, the conformation of the HA1 subunit, the main location of antigenic determinants of hemagglutinin and its recombinant monomer HA0, was not significantly affected. 

To validate our assumption, the present work aimed to study the immunogenicity and conformation of the recombinant HA1 subunit of Influenza A H1N1 hemagglutin (A/California/04/2009(H1N1)) alone and in the content of TI-complexes comprising MGDGs isolated from different marine macrophytes (*Ulva lactuca*, *Sargassum pallidum*, *Zostera marina)*, since the physicochemical properties of MGDGs depend on the taxonomy position of the marine macrophytes species of origin [13,14].

## 2. Results

### 2.1. Effect of MGDGs from Different Marine Macrophytes on the Anti-HA1 Response

**Figure 1:**
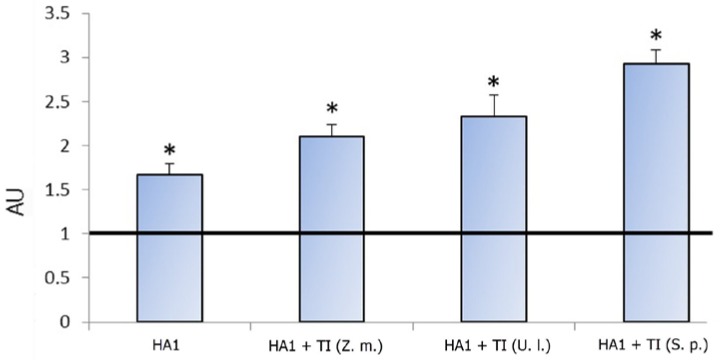
The content of anti-HA1 antibodies depending on the monogalactosyldiacylglycerol (MGDG) source. Antibodies were evaluated by ELISA in mice blood serum after two rounds of immunization by HA1 alone, HA1 incorporated in TI-complexes based on monogalactosyldiacylglycerol (MGDG) from *Zostera marina (*HA1 + TI (*Z. m.*)), *Ulva lactuca* (HA1 + TI (*U. l*.)), or *Sargassum pallidum* (HA1 + TI (*S. p*.)) at doses of 0.1 µg/mouse, administered subcutaneously at an interval of 14 days. The experiment was terminated 28 days after the first immunization. Control-mice were injected with phosphate-buffered saline (PBS). Each experimental group included six mice. The dilution of mice serum was 1/100. Data are expressed in arbitrary units (AU) relative to the control value equal to 1. Results are expressed as means ± SD of six observations. * *p* < 0.05 as compared with the control.

We used the same MGDG samples that were applied earlier in the study of secreted uncleaved HA (HA0s) of influenza A virus H1/N1 A/California/04/2009 [11]. MGDGs isolated from green algae *U. lactuca*, brown algae *S. pallidum*, and seagrass *Z. marina* differ in their fatty acid composition [13] and, therefore, microviscosity [10]. As shown in Figure 1, HA1 alone was immunogenic. It induced a 1.7-fold higher level of anti-HA1 antibodies compared with the control. In turn, all of the studied TI-complexes stimulated a 1.3–1.8 times higher immune response to HA1 compared with HA1 alone. The TI-complex based on MGDGs from *S. pallidum* showed the highest adjuvant activity compared with the TI-complexes based on MGDGs isolated from *U. lactuca* and especially from *Z. marina*.

### 2.2. Effect of MGDGs from Different Marine Macrophytes on the Cytokine Profile

**Figure 2:**
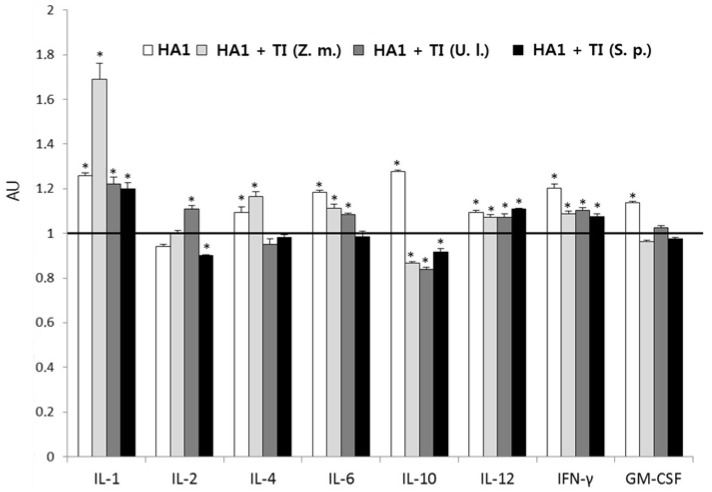
The content of cytokines depending on the monogalactosyldiacylglycerol (MGDG) source. The content of cytokines was evaluated as described in the legend of Figure 1. Data are presented in arbitrary units (AU) relative to the control value equal to 1. Results are expressed as means ± SD of six observations. * *p* < 0.05 as compared with the control.

The cytokine profile was analyzed simultaneously with analysis of the anti-HA1 antibody content. HA1 alone stimulated the production of most cytokines, except interleukine (IL)-2, whose level was somewhat lower compared with the control (Figure 2). The largest increase (about 1.3 times) was observed in the content of IL-1 and IL-10. The incorporation of HA1 in TI-compexes mainly resulted in different effects on the level of cytokines, depending on the MGDG component. All TI-complexes stimulated the production of IL-1, IL-12, and interferon-γ (IFN-γ) compared with the control. All TI-complexes, except the one based on MGDG from *S. pallidum*, also stimulated the formation of IL-6. However, the effect of HA1 on the content of TI-complexes was usually less than the effect of HA1 alone, except for HA1 incorporated in a TI-complex based on MGDG from *Z. marina*, which promoted a 1.7-fold increase in the production of IL-1 compared with the control. In addition, TI-complexes based on MGDGs from *U. lactuca* and *Z. marina* induced the synthesis of IL-2 and IL-4, respectively, more effectively than HA1 alone. 

### 2.3. Effect of MGDGs on HA1 Conformation

#### 2.3.1. Differential Scanning Calorimetry (DSC)

**Figure 3:**
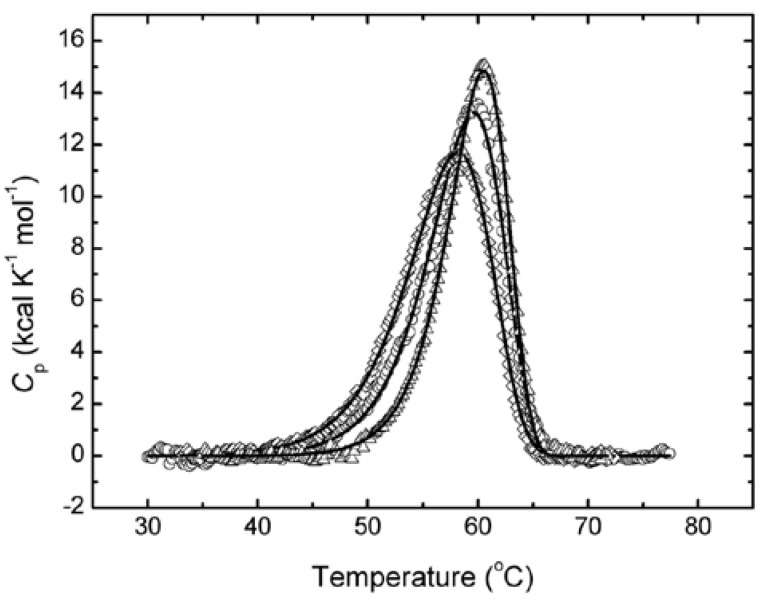
Temperature dependence of the excess molar heat capacity of the HA1 subunit of influenza A virus H1/N1 hemagglutinin alone (circles) and in the complex with monogalactosyldicylglycerol (MGDG) from *Sargassum pallidum* (squares) or *Zostera marina* (triangles) at a scan rate of 60 K h^−1^ in PBS, pH 7.4. Solid lines represent the best fit to each experimental curve using equation (2).

The functions of proteins depend on their conformation, which may change under the influence of their surroundings. Since MGDGs serve as a lipid matrix for the protein antigen, the conformation of HA1, depending on MGDGs isolated from different marine macrophytes, was studied by DSC, which allows for the evaluation of integral conformational changes in proteins by their thermal denaturation. The thermal denaturation of HA1 at pH 7.4 showed that the respective thermal transition is an irreversible, kinetically controlled process. Therefore, the resulting thermograms of HA1 alone and in the complex with MGDG from *Z. marina* or *S. pallidum* (Figure 3) were analyzed on the basis of a simple two-state irreversible model:

(1) $$N\overset{k}{\to }D$$

where N is a native HA1 and D is a denatured HA1, and *k* is a first-order denaturation rate constant that changes with temperature according to the Arrhenius equation. Thus, the excess heat capacities *C_p_^ex^* were analyzed by non-linear least-squares, fitting the data to the following equation [15]:

(2) $$C_{p}^{ex}=\frac{1}{\nu }\Delta H\exp\left\{\frac{E_{A}}{R}\left(\frac{1}{T^{*}}-\frac{1}{T}\right)\right\}\times \exp\left\{-\frac{1}{\nu }\int_{T_{o}}^{T}\exp\left[\frac{E_{A}}{R}\left(\frac{1}{T^{*}}-\frac{1}{T}\right)\right]dT\right\}$$

where ν = d*T*/d*t* (K min^−1^) is the scan-rate; Δ*H* is the enthalpy difference between the denatured and native states; *E_A_* is the activation energy of the denaturation process; *R* is the universal gas constant; and T* is the temperature at which *k* is equal to 1 min^−1^. The results of the fit are shown in Figure 3 (solid lines) and Table 1. The goodness of the fit was estimated by the correlation coefficient (*r*), calculated as:

(3) $$r=\sqrt{1-\sum_{i=1}^{n}{\left(y_{i}-y_{i}^{calc}\right)}^{2}/\sum_{i=1}^{n}{\left(y_{i}-y_{i}^{m}\right)}^{2}}$$

where *y_i_* and *y_i_^calc^* are the experimental and calculated values of *C_p_^ex^*, respectively; *y_i_^m^* is the mean of the experimental values of *C_p_^ex^* and *n* is the number of points. 

Similar to the different effects of the studied MGDGs on the level of anti-HA1 antibodies, the activation energy of the HA1 thermal denaturation (*E_A_*) essentially depended on the glycolipid surroundings (Table 1). The MGDG from *S. pallidum* caused a decrease in *E_A_*, which indicates the relaxation of the tertiary protein structure. Conversely, the more viscous MGDG from *Z. marina* [10] induced an increase in *E_A_* compared to the *E_A_* of HA1 alone and, therefore, promoted a more dense packing of HA1. The same trend occurred in the changes of T*.

#### 2.3.2. Intrinsic Fluorescence

**Figure 4:**
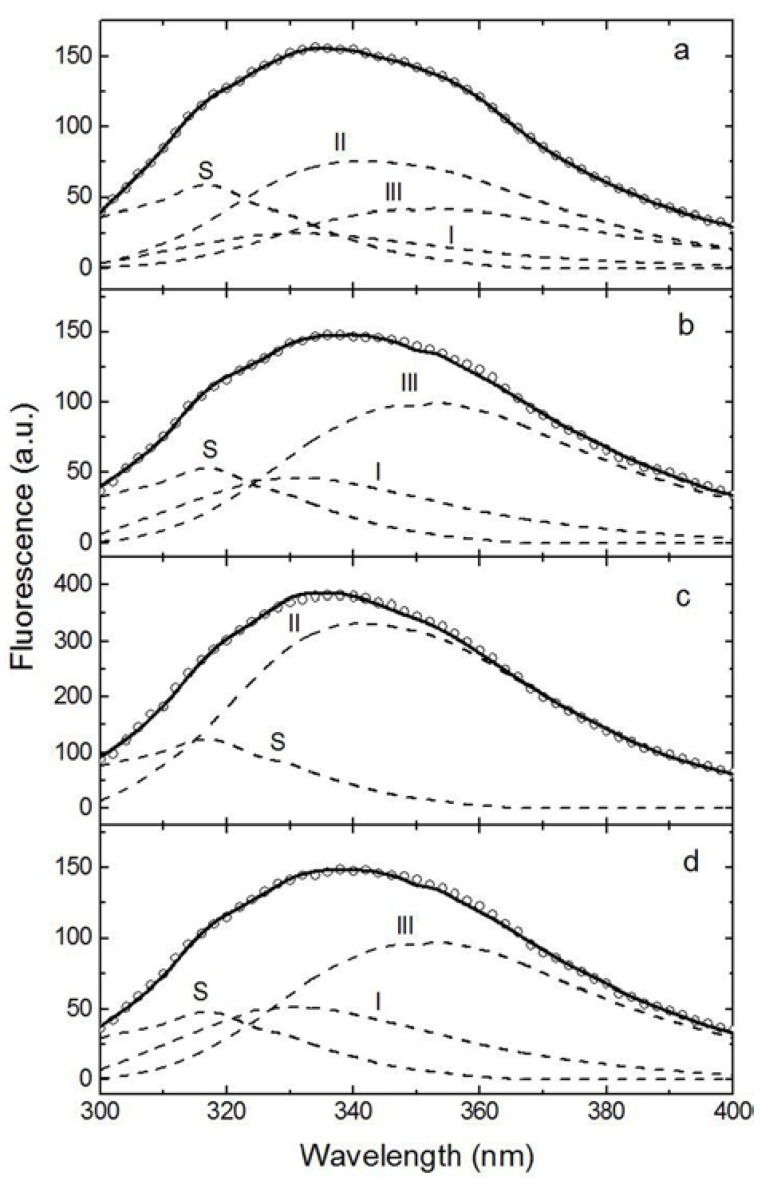
Intrinsic fluorescence spectra (symbols) of HA1 alone (**a**) and in complex with the monogalactosyldicylglycerol (MGDG) from *Sargassum pallidum* (**b**), *Zostera marina* (**c**), or *Ulva lactuca* (**d**) in PBS, pH 7.4, and their fit to the theoretical model of discrete states of tryptophan residues in proteins [16] (solid lines) which are the sums of the spectral components S, I, II, and III (dashed lines). The protein concentration was 0.05 mg/mL. The excitation wavelength was 296 nm.

A more detailed analysis of the HA1 conformation changes was carried out by intrinsic protein fluorescence. The results of deconvolution of experimental spectra into elementary components corresponding to the emission of protein fluorophores (tryptophan) [16] are presented in Figure 4 and Table 2. According to the model of discrete states of tryptophan residues in proteins, spectral forms S and I correspond to the emission of indole chromophores localized in the protein and forming exciplexes 1:1 and 2:1, respectively, with some immediate polar protein groups. Spectral form II corresponds to the emission of indole chromophores in contact with molecules of bound water at the surface of the protein. Spectral form III corresponds to the emission of an indole chromophore localized on the surface of the protein in contact with molecules of freely relaxing water. All four spectral forms, present at a ratio of 20:14:41:25, were identified upon the decomposition of the experimental spectrum of HA1 alone.

The similar microviscosity of MGDGs from *S. pallidum* and *U. lactuca* [10] resulted in their close effects on the contribution of spectral forms in HA1. Particularly strong changes occurred in the contribution of spectral forms II and III. The percentage of spectral form II dropped from 41% in HA1 alone to zero, primarily due to more than a twofold increase in the contribution of spectral form III. Far fewer changes occurred in the contribution of spectral form I, which increased by 9% and 13% compared to the contribution of this spectral form in the experimental spectrum of HA1 alone, respectively. Essentially no changes were observed from the contribution of spectral form S under the influence of all studied MGDGs. The effects of MGDG from *Z. marina* on other forms differed drastically. This glycolipid sample induced the drop in the content of spectral forms I and III to zero and, conversely, an almost twofold increase in the percentage of spectral form II. These rearrangements in the tertiary protein structure correlated with integral changes in the conformation of HA1 under the influence of MGDGs from *S. pallidum* and *Z. marina* (Figure 3, Table 1).

## 3. Discussion

The World Health Organization (WHO) recommends vaccination as the main strategy for fighting influenza [17]. HA and its HA2 and especially HA1 subunits attract much attention as promising antigens for the design of recombinant subunit vaccines against influenza. Modern genetic engineering allows obtaining HA proteins in bacterial expression systems and more rapidly producing subunit recombinant influenza vaccines, compared to traditional egg-based vaccine production techniques [18], which is especially important in a pandemic. Most recombinant proteins are weak immunogens; further studies should therefore focus on the choice of effective adjuvants. We studied the adjuvant potential of a novel antigen delivery system, TI-complexes, in relation to recombinant HA1 subunit of influenza A H1N1 hemagglutin (A/California/04/2009(H1N1)). The HA1 subunit comprises the immunodominant epitopes, most of which are conformational [8]. Hence, the immunogenicity of HA1 should depend on its spatial structure [8], which may be created by the proper glycolipid surroundings of the HA1 antigen incorporated in a TI-complex [10]. Immunization of mice with HA1 alone, and HA1 incorporated in TI-complexes based on MGDGs isolated from different marine macrophytes, revealed an immunogenicity of HA1 that confirms literature data [19,20]. The incorporation of an antigen in TI-complexes enhanced the production of anti-HA1 antibodies by 1.3–1.8 times compared with HA1 alone, depending on the physicochemical properties of the employed MGDGs [10]. The effect of MGDG from *S. pallidum*, which possesses medium microviscosity, was the highest. In turn, the most viscous MGDG from *Z. marina* provided the lowest antibody level, which principally coincides with results of the previous study on the porin from *Y. pseudotuberculosis* [10] and permits the conclusion that the microviscosity of MGDG is more important for the adjuvant effect of TI-complexes than the fatty acid composition of this glycolipid per se. The physical state of the glycolipid surroundings is likely necessary for the proper presentation of antigenic determinants of both of HA1 and porin. Unlike these results, a previous study of uncleaved HA (HA0_s_) of the same influenza A strain did not reveal any dependence of the HA0_s_ immunogenicity on the microviscosity of MGDGs. A different behavior of HA1 and HA0_s_ under the influence of studied MGDGs may be due to the direct contact of HA1 alone with the glycolipid, whereas HA1 within HA0_s_ interacts with MGDG indirectly, through a subunit HA2.

The influence of adjuvants on the cytokine profile, indicating the balance between Th1 and Th2 responses, is of great interest because of the importance of different Th cell subsets for the generation of protective immunity against infectious diseases [21]. Adjuvants can also contribute to the initiation of the innate immune response, which is characterized by cytokines (IL-1, tumor necrosis factor, and IL-6) mainly produced by macrophages and dendritic cells. As shown, cytokine profiles also were dependent on the MGDG source. If HA1 alone stimulated the biosynthesis of all studied cytokines except IL-2, HA1 incorporated in TI-complexes mainly promoted the production of IL-12 and IFN-γ secreted by Th1 cells, which mediate effective immunity against intracellular pathogens, in particular viruses [22]. HA1 incorporated in TI-complexes based on MGDG from *Z. marina* induced a sharp increase in the level of IL-1, which plays a central role in the regulation of immune responses to infections*.* Different changes in the content of other cytokines also depended on the MGDG source. 

A DSC study, which allows for the detection of integral changes in biological macromolecules, showed the opposite effect of the most rigid and the most fluid MGDGs from *Z. marina* and *S. pallidum*, respectively, on the HA1 conformation. The MGDG from *Z. marina* induced the essential increase of *E_A_* and, therefore, strong stabilization of the protein conformation, unlike the MGDG from *S. pallidum*, which provided a relaxation of the HA1 space structure. Deconvolution of the experimental fluorescence spectra of HA1 revealed a more profound effect of the MGDG from *Z. marina* on the distribution of spectral forms of the tryptophan residues compared to the effect of the MGDG from *S. pallidum.* The relaxation effect of the last MGDG sample correlated with more than a twofold increase in the contribution of the most superficial spectral form III, primarily due to the abrupt decrease in the percentage of the deeper spectral form II to zero compared with HA1 alone. The MGDG from *U. lactuca* induced similar changes in the tertiary structure of HA1. Nevertheless, the relatively slight differences in the contribution of spectral forms I and III that result from the effects of MGDGs from *S. pallidum* and *U. lactuca* indicate a denser packaging of the protein molecule under the influence of the MGDG from *U. lactuca*. In turn, the MGDG from *Z. marina* resulted in an almost twofold increase in the contribution of the spectral form II due to the decrease in the percentages of both spectral forms I, and especially III, to zero. Hence, the most relaxing effect of the MGDG from *S. pallidum* likely provides the optimal presentation of the antigen to immunocompetent cells and, therefore, the highest production of anti-HA1 antibodies.

The obtained results provide the basis for a further search for approaches to the design and optimization of the HA1 subunit-based vaccine construction.

## 4. Materials and Methods 

### 4.1. Protein Antigen

The secreted recombinant HA1 subunit of Influenza A H1N1 (A/California/04/2009(H1N1)) HA, purchased from Sino Biological Inc., Beijing, China, is the result of the expression of a DNA sequence encoding N-terminal segment (Met1-Arg344) of HA in human cells. The protein is terminated by a C-terminal polyhistidine tag. The predicted N-terminus is Arg 18. HA1 comprises 338 amino acids with a predicted molecular mass of 38 kDa. As a result of glycosylation, it migrates as an approximately 50–60 kDa band in SDS-PAGE under reducing conditions.

### 4.2. Marine Macrophytes and Preparation of MGDG

The same MGDG samples as those employed in Reference [11] were applied. To prepare them, three species of marine macrophytes from three divisions: *S. pallidum* (Phaeophyta), *U. lactuca* (Chlorophyta), and *Z. marina* (Embryophyta) were harvested in the Sea of Japan in summer at a seawater temperature of 20–23 °C. Freshly harvested algae and seagrass were heated for 2 min in boiling water to inactivate enzymes. MGDG samples were isolated from marine macrophytes as described in Reference [13]. The microviscosity of MGDG samples was determined by the method of lateral diffusion of the fluorescent probe pyrene [23] as described in Reference [10].

### 4.3. Preparation of MGDG-HA1 Samples for DSC and Spectroscopic Studies

Samples for DSC and spectroscopic studies were prepared as described in Reference [11]. MGDG dissolved in chloroform was introduced into standard aluminum pans. Vacuum-dried lipid samples of 1 mg were vortexed in 100 μL of PBS, pH 7.4, and preheated to approximately 50 °C. Then, 0.5 mg of HA1 solubilized in 0.5 mL of PBS, pH 7.4, was added and mixed with the lipid dispersion by vortex. The resulting suspension was left for 2 h and was used to study protein conformation. Protein incorporation was estimated after centrifugation of the suspension at 120,000×*g* at 4 °C for 2 h in an Optima L-90 ultracentrifuge (Beckman Coulter Inc., Brea, CA, USA). The supernatant was discarded to determine the unbound protein. Protein incorporation was about 95%.

### 4.4. Preparation of HA1-Containing TI-Complexes

HA1-containing TI-complexes were prepared according to Reference [10]. Cholesterol and MGDG dissolved in chloroform were mixed at ratio of 2:4 by weight. Then, chloroform was evaporated off under a vacuum, and six portions by weight of cucumarioside A_2_-2 isolated from the marine invertebrate *C. japonica* (Echinodermata) were added according to Reference [24]. The mixture was solubilized, and the total concentration of lipids (cholesterol and MGDG) was adjusted to 2 mg/mL of suspension in PBS, pH 7.4. The TI-complexes were mixed with 75 μg of HA1 in PBS, pH 7.4. The mixture was vortexed for 1 min and left for 2 h at room temperature. Protein incorporation was estimated as described in the previous section. Protein incorporation was about 97%. 

### 4.5. Animals and Immunization

Immunization was carried out as described in Reference [10]. Adult BALB/c mice (males) with a weight of 18–20 g were obtained from the “Puschino” nursery of laboratory animals, a branch of the Institute of Bioorganic Chemistry, RAS. The animals were kept in a vivarium under standard conditions with unlimited access to food and water according to the rules accepted by the European Convention for the Protection of Vertebrate Animals Used for Experimental and Other Scientific Purposes (Strasbourg, France, 1986). The research was carried out according to the rules of proper laboratory practices (GLP), the Order of Ministry of Health of Russian Federation 267 of 19.06.2003 “To the approval of laboratory practice regulations”, a guide to experimental (preclinical) studies of new pharmacological substances (2005). The mice were divided into five experimental groups (six animals in each): (1) mice injected with PBS (control); (2) mice immunized with individual HA1; (3–5) mice immunized with HA1 incorporated into TI-complexes based on MGDGs from *U. lactuca*, *S. pallidum*, or *Z. marina*, respectively. The mice were immunized subcutaneously twice, applying a dose of 0.1 μg of HA1 per mouse, at an interval of 14 days. The experiment was terminated 28 days after the first immunization.

### 4.6. ELISA

Blood sera of the experimental mice were obtained to determine the content of the anti-HA1 antibodies and cytokines. The blood of the mice was collected immediately after decapitation. The blood was incubated at 37 °C for 2 h. After clot retraction, the samples were centrifuged at 1500 rpm for 10 min. The supernatant was taken into plastic tubes and stored at −20 °C.

The content of anti-HA1 antibodies was estimated in mouse blood serum by ELISA, applying anti-mouse IgG labeled with peroxidase (Research Institute of Epidemiology and Microbiology, RAMS) as described in Reference [11]. The sera of the PBS-injected animals served as negative controls. Sensibilization of solid surface was carried out by the introduction of HA1 solution into wells of a 96-well microtiter plate (GosNIIMedPolimer, Russia) (1 μg of HA1 in 0.1 mL carbonate bicarbonate buffer, pH 9.6 per well). The optical density of the antibody samples was estimated using an Elx808IU microplate photometer (BioTek Instruments, Inc., Winooski, VT, USA) at a wavelength of 450 nm (chromogen - 3,3′,5,5′-tetramethylbenzidine; BD, USA). Data is expressed in arbitrary units (AU) relative to the control value equal to 1. The dilution of the serum was 1/100. Cytokines (interleukin (IL)-1, IL-2, IL-4, IL-6, IL-10, IL-12, IFN-γ, and GM-CSF were determined in the blood sera of mice from each group by noncompetitive ELISA using BD OptEIATM Mouse ELISA SET (BD). Absorption was recorded with an Elx808IU microplate photometer (BioTek, Instruments, Inc.) at a wavelength of 450 nm. Results are expressed as means ± SD of six observations. Differences among the means were analyzed by parametric analysis using Student’s *t*-test. *p* < 0.05 was considered to be statistically significant.

### 4.7. Calorimetry

Calorimetric experiments were performed on a Scal-1 differential scanning microcalorimeter (Scal Co. Ltd., Pushchino, Russia) as described in Reference [10]. The data were processed by SCAL software. The resulting temperature dependencies of the partial molar heat capacity were further analyzed and plotted using homemade software [15] as well as a Windows-based software package (ORIGIN) supplied by MicroCal.

### 4.8. Fluorescence Spectroscopy

Steady-state fluorescence measurements were carried out on a PC1 spectrofluorimeter (ISS, Illinois, USA) at 25 °C as described in Reference [10]. The excitation wavelength was 296 nm. The monochromator slit width was kept at 5 nm in the excitation and emission channels. Fluorescence was measured in the range of 300–400 nm. Fluorescence measurements were carried out in protein solutions with an optical density of less than 0.2 at 280 nm in order to avoid the inner filter effect. Emission spectra were corrected for baseline and instrumental spectral sensitivity.

## Figures and Tables

**Table 1:** Arrhenius equation parameter estimates of the thermal denaturation of HA1 alone and in complexes with MGDGs from different marine macrophytes.

Sample	*E_A_* ^1^ (kcal/mol)	T* (K) ^2^	*r* ^3^
HA1	65.7 ± 1.3	332.6 ± 0.3	0.9999
HA1 + MGDG from *S. pallidum*	57.5 ± 1.2	331.3 ± 0.2	0.9998
HA1 + MGDG from *Z. marina*	81.7 ± 1.4	334.1 ± 0.4	0.9999

^1^ Experimental energy of activation of the denaturation process. ^2^ Temperature when the denaturation rate constant (*k*) equals 1 min^−1^. ^3^ The correlation coefficient. ^1,2^ The data are expressed as the mean ± average deviation of three separate determinations.

**Table 2:** Influence of monogalactosyldicylglycerol (MGDG) from marine macrophytes on the contribution of tryptophan spectral forms to the total fluorescence spectrum of HA1, %.

Sample	Spectral Forms			
	S (λ_max_ = 317 nm)	I (λ_max_ = 332 nm)	II (λ_max_ = 342 nm)	III (λ_max_ = 353 nm)
HA1	20 ± 0.4	14 ± 0.1	41± 0.3	25 ± 0.3
HA1 + MGDG from *S. pallidum*	19 ± 0.3	23 ± 0.2	0	58 ± 0.4
HA1 + MGDG from *Z. marina*	21 ± 0.4	0	79 ± 0.6	0
HA1 + MGDG from *U. lactuca*	18 ± 0.2	27 ± 0.3	0	55 ± 0.5

The data are expressed as the mean ± average deviation of three separate determinations.

## Abbreviations

HA	Hemagglutinin
TI-complex	Tubular immunostimulating complex
MGDG	Monogalactosyldiacylglycerol
ELISA	Enzyme-linked immunosorbent assay
DSC	Differential scanning calorimetry
GM-CSF	Granulocyte-macrophage colony-stimulating factor
IL	Interleukin
IFN	Interferon
PBS	Phosphate-buffered saline
SDS-PAGE	Sodium dodecyl sulfate polyacrylamide gel electrophoresis
